# Catastrophic Thromboembolic Syndrome During the COVID-19 Pandemic

**DOI:** 10.7759/cureus.16854

**Published:** 2021-08-03

**Authors:** Ndausung Udongwo, Asseel Albayati, Arda Akoluk, Amanda Woodford, Jose Iglesias, Deepak Singh

**Affiliations:** 1 Internal Medicine, Jersey Shore University Medical Center, Neptune City, USA; 2 Nephrology, Hackensack Meridian School of Medicine at Seton Hall University, Nutley, USA; 3 Cardiothoracic Surgery, Jersey Shore University Medical Center, Neptune City, USA

**Keywords:** covid19, hypercoagulability, thrombosis, catastrophic thrombosis syndrome, aortic thrombus

## Abstract

Since March 2020, the SARS-CoV-2 virus known to cause COVID-19 has presented in many ways and it seems to affect almost all organ systems. One of its detrimental effects is on the coagulation system. Disruption of the coagulation pathway or hypercoagulability has been reported extensively in many articles and studies. It seems there is no specific pattern or location of the coagulopathy. The coagulopathy can present as part of the respiratory disease process or as an isolated phenomenon. Many articles had reported that the thrombus can be a venous thromboembolic phenomenon such as deep venous thrombosis (DVT), portal vein thrombosis, pulmonary embolism (PE), or as arterial thrombosis, for instance, coronary artery thrombosis, cerebrovascular thromboembolic disease (i.e., stroke), or an aortic thrombus. One of the disastrous presentations is what is called “catastrophic thrombosis syndrome.” This syndrome is characterized by multiple thromboses that take place in different parts of the vascular system at different parts of the body at the same time. In many studies, D-dimer levels have been shown to predict the risk of increased thromboembolism in SARS-CoV-2. However, an appropriate anticoagulation agent, dosage, and duration are yet to be determined. We are presenting an interesting case of a female who suffered catastrophic thrombosis syndrome despite being on prophylactic anticoagulation. She presented with leg pain and was found to have extensive multiple thrombi starting from the ascending/descending aorta and extending to the distal peroneal arteries.

## Introduction

The coronavirus disease 2019 (COVID-19) pandemic affected patients in many ways across the globe. As of early 2021, there have been more than one million global deaths reported by the World Health Organization (WHO) [1]. Almost all COVID-19 patients demonstrated coagulopathy and thrombosis [2]. As a result, current guidelines recommend at least prophylactic anticoagulation, if not full dose anticoagulation in severe cases [3]. Despite the current guidelines, many cases of patients with anticoagulation present with embolism and thrombosis [3-5]. We present a case of a 63-year-old female with a recent COVID-19 disease that was anticoagulated but presented with thrombotic events that involved the left atrial appendage, the distal ascending, and descending thoracic aorta, distal abdominal aorta, right common femoral artery, posterior tibial, and peroneal arteries.

## Case presentation

**Figure 1:**
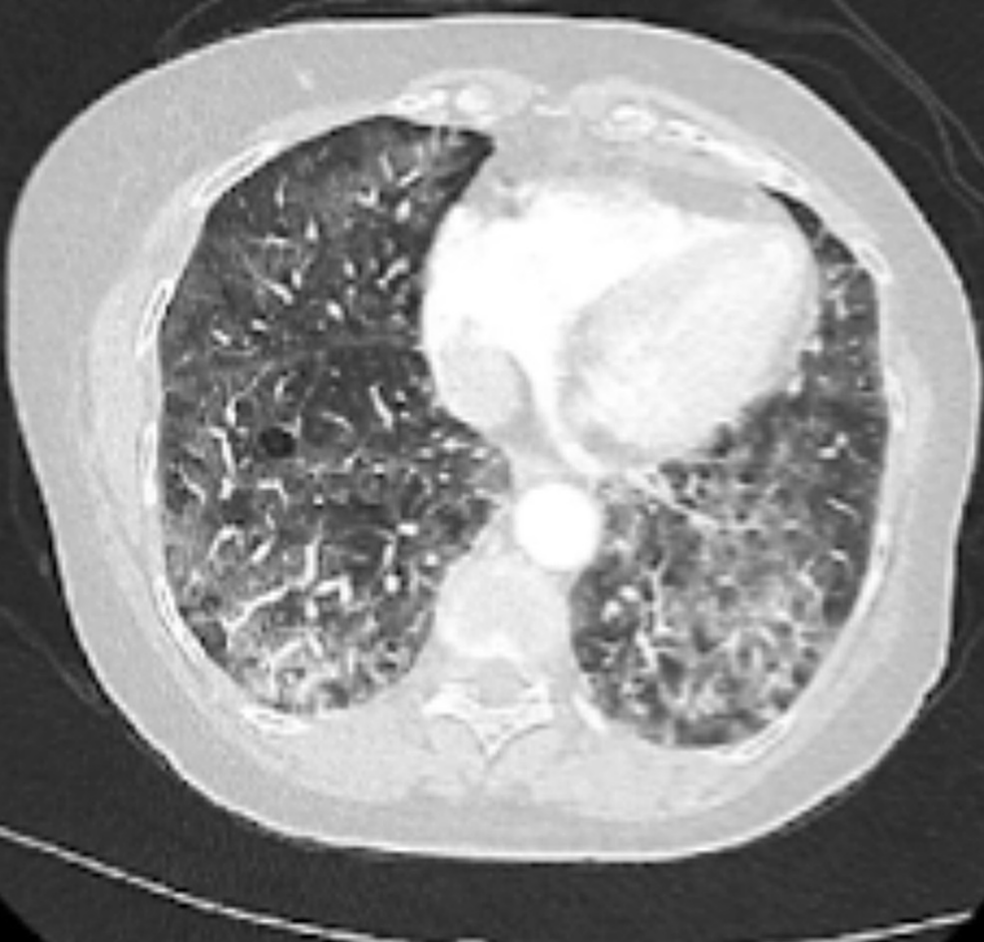
CT angiogram of the chest showing advanced centrilobular emphysema with nonspecific thickening of the pulmonary septa and associated ground-glass opacity predominating the mid to lower lung field.

A 63-year-old female presented initially with a history of seven days diarrhea, three days of shortness of breath, and pleurisy. Her past medical history was significant of remote stage 1 breast carcinoma status post-mastectomy, COPD, and ex-smoker. Physical examination was significant only for decreased air entry bilaterally with coarse crackles. Chest x-ray (CXR) and computed tomography angiogram (CT-A) of the chest confirmed bilateral ground-glass opacities of mid to lower lung fields without evidence of pulmonary embolism (PE) (Figure 1). SARS-COV-2 PCR test was positive. On presentation, her oxygen saturation (SpO_2_) was 88% on room air but improved on nasal cannula. D-dimer was normal at 483 ng/mL (ref range ≤500 ng/mL), troponin was 0.01, and procalcitonin was negative. She was started on dexamethasone 6 mg PO daily for 10 days, remdesivir 100 mg/250 mL intravenous infusion daily for five days, enoxaparin 40 mg subcutaneously daily for deep venous thrombosis (DVT) prophylaxis. On the 10th day of her symptoms, the D-dimer suddenly spiked to 2,080 ng/mL and her enoxaparin dose was increased to 40 mg subcutaneously every 12 hours. At that point, the patient finished five days of remdesivir, she was ambulating without shortness of breath, and her SpO_2_ was >90% on room air. She was discharged home on dexamethasone 6 mg PO daily for five more days and started rivaroxaban 10 mg PO daily for 30 days. A follow-up visit was scheduled in one week with her primary care physician.

In less than 24 hours of discharge, the patient presented to the emergency department complaining of severe right foot pain and cramping that started two hours prior. She also reported chest tightness and shortness of breath. The sharp/shooting pain was severe enough in nature that it would limit ambulation. Her SpO_^2^_ on room air was 84% but improved to 94% when placed on OptiFlow with a flow rate of 50 liters and FiO_^2^_ of 70%. Otherwise vitally stable and examination was unremarkable. Her blood work is illustrated in Table 1.

**Table 1:** Laboratory results of the patient

Test	Result	Reference range
WBC	5.1 x 10^ 3^/µL	4.5–11.0 x 10^3^/µL
Hemoglobin	16.6 g/dL	12–16 g/dL
Platelet	307 x 10^3^/µL	140–450 x 10^3^/µL
international normalized ratio (INR)	1.5	0.9–1.2
Partial prothrombin time	34 seconds	26–39 seconds
D-dimer	5,559 ng/mL	≤500 ng/mL
interleukin 6	14.3 pg/mL	≤2 pg/mL
lactate dehydrogenase	564 U/L	91–200 U/L
Ferritin	1,012.2 ng/mL	11–307 ng/mL
Anticardiolipin Ab IgG	7 GPL	0–14 GPL
Beta-2 glycoprotein IgG Ab	5 SGU	0–20 SGU
Beta-2 glycoprotein IgM Ab	11 SMU	0–20 SMU
Beta-2 glycoprotein IgA Ab	12 SAU	0–20 SAU
Dilute Russell viper venom (Lupus anticoagulant)	1.0	Ratio 0.8–1.2 lupus anticoagulant
B-type natriuretic peptide	24 pg/mL	100 pg/mL
C-reactive protein	1.51 mg/dL	0.00–0.74 mg/dL
JAK2 V617F mutation detection	Not detected	Not detected

**Figure 2:**
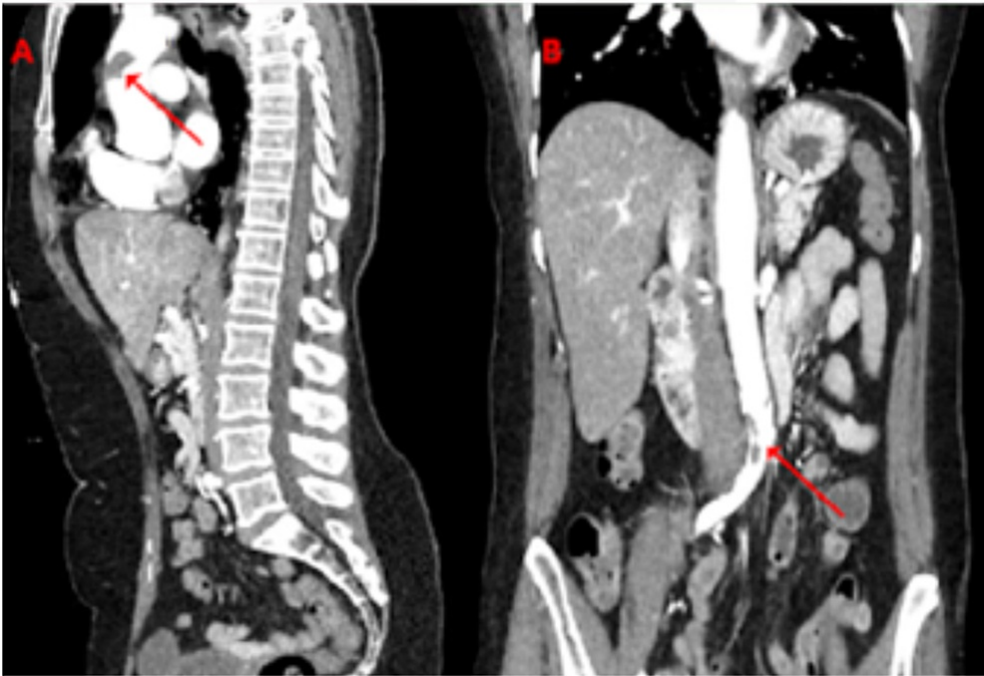
Computerized tomography with contrast angiography of the abdominal aorta with bilateral iliofemoral runoff (A) sagittal view, a large focus of free-floating thrombus in the ascending thoracic aorta (arrow). (B) Coronal view, a large focus of thrombus in the distal abdominal aorta that extends into the origin of the right common iliac artery (arrow).

Meanwhile, the patient’s CXR was similar to previous studies without acute changes. Bilateral lower extremity duplex doppler ultrasound showed diminished velocity in the right popliteal artery and right posterior tibial artery suggesting diminished inflow. The anterior tibial artery was patent. Immediately the patient had a CT angiography of the abdominal aorta with bilateral iliofemoral runoff that showed a large free-floating thrombus in the ascending aorta with few scattered eccentric thrombi in the descending and infrarenal abdominal aorta. In addition, there was a large thrombus in the distal abdominal aorta that extended into the origin of the right common iliac artery resulting in short segment severe stenosis. The remainder of the iliofemoral and popliteal segments were patent without hemodynamically significant stenosis. There was also an abrupt cut-off in the posterior tibial and peroneal arteries in the mid-calf on delayed phase images suggesting distal embolization (Figures 2A, 2B).

The general surgery, vascular surgery, and cardiothoracic teams recommended systemic anticoagulation without surgical intervention. The patient was started on high molecular weight heparin infusion at 14 units/kg/hr. and was closely monitored in the intensive care unit. After monitoring for 24 hours in the intensive care unit with serial neurological and vascular examination with Doppler ultrasound, the patient was downgraded to the telemetry unit. After three days, the patient remained clinically stable, and her oxygen requirement lowered gradually to room air. Repeat CT angiography of the abdominal aorta with bilateral iliofemoral runoff showed stable foci of thrombus compared to the previous study. The patient was continued on heparin infusion and was being transitioned to warfarin orally daily with an international normalized ratio (INR) goal of 2.5-3.5. After nine days, the patient was medically and surgically stable. Her INR was at goal (2.61) and discharged on warfarin 2.5 mg tablets orally daily with outpatient follow-up for INR monitoring.

The patient was being followed up as an outpatient and she remained clinically stable. After five months, repeat CT angiography of the chest, abdomen, and pelvis showed complete resolution of the ascending and descending thoracic and abdominal intraluminal aortic thrombi. No aortic aneurysm or dissection.

## Discussion

COVID-19 is associated with an increased risk of thromboembolic incidents, and some mechanisms described in the literature may be involved in the pathophysiology of these complications [2]. This could be multifactorial involving endothelial injury, increased circulation of proinflammatory cytokines (causing increased activation of polymorphonuclear cells, particularly neutrophils), disinhibition of platelet activity, inhibition of anticoagulants, and immobilization which is common in the ICU setting [4,5].

Severe COVID-19 infection increases the activation of neutrophil extracellular traps (NETs). This process is regarded as NETosis [6]. NETs induce the expression of tissue factor (TF) resulting in increased platelet activation/thrombosis. Skendros et al. reported that increased complement activations contributed to the maladaptive immune response seen in COVID-19 patients [6]. Also, this study suggested that in order to decrease thrombosis in COVID-19 patients, treatment should be aimed to target NETs formation and complement activation [6].

The American Society of Hematology recommends that “all patients hospitalized with COVID-19 infection, should receive thromboprophylaxis with LMWH or fondaparinux” [7].

Most of the reports on a high incidence of thrombotic complications are in relation to deep venous thrombosis (DVT) and pulmonary embolism (PE) [8], while the evidence about arterial thrombosis in patients with COVID-19 is limited. To our knowledge, thoracic aortic mural thrombus (TAMT) that is free-floating is an uncommon manifestation in SARS-CoV-2 infection. Also, there are only a few cases in literature with similar presentations. These are summarized in Table 2 including their presentations and various laboratory results (D-dimer levels) correlating to increased risk of this disease. Thrombotic complications tend to occur later in the disease, at least 5 days after their presentation. D-Dimer seems to be the best laboratory marker for a possible prothrombotic state. Conservative treatment seems appropriate initially if there are no distal emboli.

**Table 2:** A comparative study showing the differences in biographical, clinical, biochemical, and treatment modalities among patients affected by COVID-19-related thrombosis. Abbreviations: M: Male, F: Female, HTN: Hypertension, DM: Diabetes, HLD: dyslipidemia, HMWH: High molecular weight heparin, LMWH: Low molecular weight heparin

	Age (Years) and Sex	Risk factors	Thrombus characteristics	Thrombus treatment	COVID-19 symptoms	D-dimer (µg/L)	LDH (µ/L)	CRP (mg/L)	Ferritin (ng/mL)	Il-6 (pg/mL)	Pneumonia characteristics	DVT/PE prophylaxis and dose	COVID-19 treatment
Our case	63, F	Tobacco	Multiple (3) floating	HMWH	8	222–5,559	NA–564	0.76–3.20	969–1,012	8.1–14.3	severe, bilateral	Rivaroxaban (10 mg daily)	Dexamethasone, remdesivir
Caranzza case 1 [9]	78, M	HLD	Multiple (3) floating	LMWH	9	910–3,570	327–361	86.8–38.6	1,510–1,272	NA–64.1	severe, bilateral	Enoxaparin (60 mg daily)	Piperacillin-tazobactam, azithromycin, hydroxychloroquine, methylprednisolone, tocilizumab
Caranzza case 2 [9]	76, M	HTN, HLD, DM type 2	Multiple (2) floating	LMWH	26	1,340 - 2,220	364–313	133.1–4.8	NA–403	NA–81	severe, bilateral	Enoxaparin (60 mg daily)	Ceftriaxone, azithromycin, hydroxychloroquine, methylprednisolone, tocilizumab
Caranzza case 3 [9]	64, M	Tobacco use, HTN	Unique, floating	HMWH followed by LMWH	11	670–4,640	169–439	81.5–10.8	NA–205	NA–NA	severe, bilateral	Enoxaparin (60 mg daily)	Ceftriaxone, azithromycin, hydroxychloroquine, methylprednisolone, tocilizumab
Gomez-arbelaez case 1 [10]	67, M	Hypertension	Occlusion	LMWH	17	7,756	893	8.1	NA	657	NA	Enoxaparin, prophylactic dose	empiric antibiotic, anti-viral treatment
Gomez-arbelaez case 2 [10]	50, M	None	Occlusion	LMWH	12	19,289	823	3.8	NA	507	NA	Enoxaparin, prophylactic dose	empiric antibiotic, anti-viral treatment
Gomez-arbelaez case 3 [10]	76, F	HTN, HLD, psoriasis	Floating thrombus	LMWH	15	1,077	391	28.6	NA	176	NA	Enoxaparin, prophylactic dose	empiric antibiotic, anti-viral treatment
Gomez-arbelaez case 4 [10]	69, M	None	Floating thrombus	LMWH	15	31,336	510	3.4	NA	1,138	NA	Enoxaparin	empiric antibiotic, anti-viral treatment
Borulu case 1 [11]	70, M	HTN, Tobacco, HLD	Occlusion	Surgical procedure	16	12,500	1,456	4.77	969,3	NA	Bilateral	None	COVID-19 treatment, favipiravir, prednisolone, levofloxacin
Borulu case 2 [11]	49, M	CAD, DM	Multiple (2) floating	Surgical procedure	13	5,890	216	2.61	687	NA	Bilateral	None	antiviral treatment
Woehl case 1 [12]	64, M	None	Floating thrombus	None	18	2,160	NA	16	NA	NA	Bilateral ground-glass opacities	NA	NA
Woehl case 2 [12]	68, M	HTN, Tobacco, HLD	Occlusion	None	10	1,696	NA	35	NA	NA	Bilateral ground-glass opacities	NA	NA
Woehl case 3 [12]	72, M	HTN, HLD, DM type 2	Floating thrombus	None	28	1,825	NA	7	NA	NA	Bilateral ground-glass opacities	NA	NA
Woehl case 4 [12]	78, M	HTN, Tobacco	Floating thrombus	None	8	4,169	NA	132	NA	NA	Bilateral ground-glass opacities	NA	NA

TAMT with no pre-existing aortic disease is a rare entity on its own, and treatment in COVID-19 infection is still debatable. In our case, after multidisciplinary discussions, we decided to move on with a conservative approach and treat medically despite the grave presentation. In literature, it is recommended that if there is any sign of ischemia, thrombectomy is warranted and has been performed successfully in COVID-19 patients [7].

Another discussion and topic of active research in COVID-19 infection is discharge anticoagulation. It is well known at this time that patients hospitalized for acute medical illness are at increased risk for venous thromboembolism (VTE) for up to 90 days after discharge [13]. Asymptomatic VTE incidence of between 0% and 0.6%, at 30-42 days post-COVID-19 discharge has been reported in observational studies in patients with COVID-19. Aspirin has been investigated for VTE prophylaxis in low-risk patients after discharge. It is also currently being investigated for post-discharge prophylaxis in COVID-19. Although our patient was treated with enoxaparin during hospitalization and discharged on a course of rivaroxaban, the ideal direct oral anticoagulant is still unclear in this patient population [14]. 

## Conclusions

COVID-19 viral infection not only affects the respiratory system. So far, we have seen many presentations of this infection. One of the common presentations is thromboembolic disease. Despite the efforts to control this disease, it still imposes difficulty on the treating physician as this disease lacks the screening tools and the definitive treatment protocols. In this case report and article review, we focused the light on one of the catastrophic presentations of thromboembolic disease in COVID-19 disease and the effectiveness of anticoagulation. Bigger case series or cohort studies on the thrombosis incidence associated with COVID-19, follow-up markers, the role of anticoagulation with the options and dosages are all essential to understanding this uncommon form of life-threatening thrombosis.
